# Plasma proteomic profiles from disease-discordant monozygotic twins suggest that molecular pathways are shared in multiple systemic autoimmune diseases*

**DOI:** 10.1186/ar3506

**Published:** 2011-11-01

**Authors:** Terrance P O'Hanlon, Zhuoyan Li, Lu Gan, Mark F Gourley, Lisa G Rider, Frederick W Miller

**Affiliations:** 1 Environmental Autoimmunity Group, National Institute of Environmental Health Sciences, National Institutes of Health, DHHS, 9000 Rockville Pike, Bethesda, MD 20892 USA; 2 National Institute of Arthritis and Musculoskeletal Disease, National Institutes of Health, DHHS, 9000 Rockville Pike, Bethesda, MD 20892 USA

**Keywords:** autoimmunity, inflammation, human, proteome

## Abstract

**Introduction:**

Although systemic autoimmune diseases (SAID) share many clinical and laboratory features, whether they also share some common features of pathogenesis remains unclear. We assessed plasma proteomic profiles among different SAID for evidence of common molecular pathways that could provide insights into pathogenic mechanisms shared by these diseases.

**Methods:**

Differential quantitative proteomic analyses (one-dimensional reverse-phase liquid chromatography-mass spectrometry) were performed to assess patterns of plasma protein expression. Monozygotic twins (four pairs discordant for systemic lupus erythematosus, four pairs discordant for juvenile idiopathic arthritis and two pairs discordant for juvenile dermatomyositis) were studied to minimize polymorphic gene effects. Comparisons were also made to 10 unrelated, matched controls.

**Results:**

Multiple plasma proteins, including acute phase reactants, structural proteins, immune response proteins, coagulation and transcriptional factors, were differentially expressed similarly among the different SAID studied. Multivariate Random Forest modeling identified seven proteins whose combined altered expression levels effectively segregated affected vs. unaffected twins. Among these seven proteins, four were also identified in univariate analyses of proteomic data (syntaxin 17, α-glucosidase, paraoxonase 1, and the sixth component of complement). Molecular pathway modeling indicated that these factors may be integrated through interactions with a candidate plasma biomarker, PON1 and the pro-inflammatory cytokine IL-6.

**Conclusions:**

Together, these data suggest that different SAID may share common alterations of plasma protein expression and molecular pathways. An understanding of the mechanisms leading to the altered plasma proteomes common among these SAID may provide useful insights into their pathogeneses.

## Introduction

Systemic autoimmune diseases (SAID) (for example, systemic lupus erythematosus (SLE), rheumatoid arthritis, scleroderma, and dermatomyositis) result in significant morbidity and mortality and a large socioeconomic burden in the United States, where they are estimated to afflict more than five percent of the population [1]. Evidence for immune-mediated pathologies associated with these heterogeneous syndromes comes from the frequent finding of autoantibodies, chronic inflammation of multiple organ systems, and clinical improvement with immunosuppressive therapy. Familial disease associations but limited disease concordance between monozygotic (MZ) twins, ethnogeographic and seasonal clustering of disease onset, and the identification of shared genetic risk factors support the hypothesis that chronic immune activation in SAID is triggered by specific environmental exposures in genetically susceptible individuals [2].

Proteomic analyses of human biological fluids (for example, plasma, urine, saliva, cerebral spinal and synovial fluids) have enabled the differential quantitation of large numbers of protein molecules between healthy and diseased subjects. Studies utilizing bio-fluid proteomics have identified multiple, pathologic markers and molecular pathways associated with different disease phenotypes, severities, and therapeutic responses [3,4]. Yet, despite these in-roads, considerable variability in the published SAID literature exists and likely results from multiple factors including different proteomic methodologies (for example, 2-D electrophoresis, mass spectrometry, antibody array), choice of bio-fluids or tissues analyzed, and the inherent heterogeneity of SAID phenotypes, patient histories, and human genetic variations. Nevertheless, some consensus has emerged in multiple, independent lines of proteomic research in the rheumatic diseases [4]. These common findings in multiple rheumatic diseases to date include Type I interferon inducible proteins, autoantibodies, numerous inflammatory cytokines/chemokines, and markers of molecular pathways associated with chronic immune activation (for example, NF-kB, TNFα, and complement fixation), oxidative stress, coagulation, protein degradation and lipid metabolism [3-8].

Proteomic analysis of blood plasma has several useful research advantages despite its technical complexity. Blood plasma has an exceedingly complex proteome consisting of approximately 1,000 distinct polypeptides, whose concentrations vary over several orders of magnitude [9]. The vast majority of total plasma protein, however, is comprised of a smaller number of more abundant proteins (for example, albumin, immunoglobulins and haptoglobin), which necessitate their pre-depletion to enhance the detection of other minor protein constituents present at much lower concentrations. Despite these methodologic challenges, the plasma proteome is one of the most extensively characterized bio-fluids in humans [10,11]. Moreover, plasma samples are more easily obtained using a minimally invasive procedure, and are an ideal source of circulating disease-associated markers as well as those derived from dead or leaking cells from pathologic tissues throughout the body [3,4,9].

In human proteomic studies, statistically significant differences in protein levels among experimental and control subjects are often subtle (that is, 1.5- to 4-fold variations) and influenced potenlially by the degree of genetic variation that exists among human study subjects [3,4,11]. To help mitigate the potentially confounding effects of human genetic polymorphisms in our study population, we utilized liquid chromatography electrospray ionization mass spectrometry (LC-ESI-MS) to measure quantitative differences in the plasma proteome of SAID-discordant MZ twins and unrelated, matched controls. In a hypothesis-generating study, we sought to compare plasma proteomes with the expectation of identifying putative disease-associated markers among study subjects with greater genetic similarity, but possibly different environmental and/or epigenetic influences. To this end, we have identified multiple molecular pathways and possible biomarkers common among different SAID.

## Materials and methods

MZ twin pairs discordant for SAID and unrelated, matched, healthy controls (*n *= 10) were identified for this study. These subjects were selected among those enrolled and providing informed consent between 2001 and 2006 in the NIH investigational review board-approved Twins-Sib study assessing the pathogenesis of SAID. Ethical approval for this proteomics study was obtained from the NIH investigational review board and all human subjects provided informed consent. Study subjects included nine Caucasian twin pairs and one twin pair of Hispanic descent. Patients were defined as those meeting American College of Rheumatology (ACR) criteria for systemic lupus erythematosus (SLE), juvenile idiopathic arthritis (JIA), or juvenile dermatomyositis (JDM) and required the exclusion of inherited, metabolic, infectious diseases or other mimics of SAID; patients were within four years of diagnosis. Twin monozygosity was confirmed by short tandem repeat analysis of genomic DNAs (Proactive Genetics Inc., Martinez, GA, USA). Study subjects comprised three groups: (1) 10 SAID probands (4 SLE, 4 JIA, and 2 JDM); (2) probands' 10 autoimmune disease unaffected MZ twins; and (3) 10 unrelated, matched controls who were also free of SAID. The 10 sets of twin pairs included 6 juveniles (mean age 12.2 years) and 4 adult cases (mean age 25.8 years). The mean ages of juvenile and adult unrelated, healthy controls were 9.8 and 27 years, respectively. Each study group had seven females and three males. Physical global disease activity assessments were determined on a visual analogue scale (0 to 100 mm): SLE (mean 13.2, range 4 to 30); JIA (mean 25.2, range 0 to 40); JDM (mean 4.5, range 2 to 7). To minimize potential confounders, plasma samples were collected in the morning with immunosuppressive therapy held at least 24 hours prior to collection. Unrelated controls were age- (within six years), gender- and ethnically-matched to twins, were free of infections, trauma, vaccines and surgeries for eight weeks and had no first degree family members with SAID.

### Proteomic differential expression analysis

Plasma samples were collected and frozen within one hour at -80°C. All samples were shipped on dry ice to PPD Inc. Biomarker Discovery Sciences (Menlo Park, CA, USA). Upon processing, thawed samples were stabilized with a sodium azide and a protease inhibitor cocktail containing 100 ug/mL aprotinin and 5% (w/v) sodium azide, which were added to the plasma at a volume ratio of 1:100. Experimental run order was prepared within a block randomization scheme consisting of matched twin and control samples (10 blocks of 3 subjects each). The order of processing and analyzing samples was separately randomized within each block. Plasma proteins were analyzed by mass spectrometric analysis using a one-dimensional (1-D) separation approach as described below [12].

For the proteomic analysis, plasma was pre-depleted for the six most abundant proteins (albumin, IgG, IgA, haptoglobin, transferrin and α1 anti-trypsin) by an antibody-based affinity column. The remaining proteins were denatured, reduced, and sulfhydryl groups carboxymethylated prior to trypsin digestion. Also, prior to the trypsin digestion, low molecular weight molecules were excluded during a buffer exchange step with a 5 kDa cut-off filter. Tryptic peptides were then profiled by liquid chromatography-electro-spray ionization-mass spectrometry (LC-ESI-MS) on a high-resolution (R > 5,000) time-of-flight (TOF) instrument (Waters Corp., model LCT) Milford, MA, USA using a capillary chromatography column. The on-line chromatography pump (Agilent, model capillary 1100) Santa Clara, CA, USA was used for reverse-phase (RP) separation with a water/acetonitrile gradient and 0.1% formic acid added to aid in ionization efficiency and chromatographic behavior. A total of 9,549 molecular components were tracked and quantified in the 1-D analysis.

Quality control samples from a large human plasma pool were chemically processed and analyzed along with the clinical samples with an average frequency ratio of one QC sample per eight clinical samples. Process quality control samples were required to maintain coefficients of variation (CV) for many endogenous biomolecules of less than 20%.

### Peptide identification

Peptides of interest (significantly different in plasma levels) were linked by accurate mass and chromatographic retenion time to separate tandem mass spectrometry (MS/MS) experiments on an ion-trap mass spectrometer (Thermo, model LTQ, West Palm Beach, FL, USA). The resulting MS/MS spectra contained fragmentation patterns with characteristic peptide backbone cleavages. Each MS/MS raw spectrum from an isolated precursor ion was compared with *in silico *protein digestion and fragmentation data using the NCBI RefSeq sequence database to find a match-quality score and subsequent identification. Mascot software from Matrix Science (Boston, MA, USA) was used for peptide identification. To help separate correct from incorrect database search results, probabilities of correct identification were computed by unsupervised machine learning with an expectation-maximization (EM) algorithm [13]. Here, the probabilities are based both on Mascot scores and on the differences between observed and predicted retention time or retention index. The retention time is predicted using amino acid composition throughout the peptide and specifically at the amino-terminus, as well as peptide length, following the approach previously published [14], but trained on a data set similar to that acquired here. In this study the probability minimum threshold was set to 0.8.

### Quantification strategy

A label-free differential quantification method was employed that relies on changes in analyte signal intensities directly reflecting their concentrations in one sample relative to another [12,15]. This quantification technology employs overall spectral intensity normalization by employing signals of molecules that do not significantly change concentration from sample to sample. A simple correction can be applied for any differences in sample concentrations and/or drift over time in LC-MS instrument response. The computation performs normalization by determining the median of the ratios for a large number of molecular ions (spectral components). Analysis of the data included spectral smoothing, baseline subtraction, noise evaluation, peak identification, intensity evaluation, inter-scan evaluation to construct chromatographic peaks and to establish molecular components, and final signal quantification [12,15]. All processed, primary data are provided as a supplementary submission to this article (Additional files 1, 2, 3).

### Statistics

If the data of the different study groups were approximately normally distributed as determined by the Shapiro-Wilk test, then a two-sided t-test was used; if not, the nonparametric rank test (Wilcoxon or Kruskal-Wallis test) was applied. These comparisons are paired for the two draw times from each individual. Fold-changes in quantitative expression and *P*-values were determined. All tests of hypotheses in this exploratory study were two-sided and a *P*-value of < 0.05 was considered significant.

As an alternative means of data interpretation, we determined the relative importance that combined sets of protein components confer upon the accurate classification of the individual study groups (affected twins, unaffected twins, and unrelated, matched controls) using the Random Forests (RF) algorithm developed by Breiman and Cutler [16,17]. The quantitative expression levels of all factors identified in the 1-D differential expression analysis of disease-discordant twin pairs were classified using RF models (decision trees = 500, node size = 3). Individual decision trees were constructed from combined, unmatched cases and control training data sets utilizing bootstrap sampling with replacement and random variable selection. Classification was performed by a majority vote across the separate trees using test cases and controls omitted from the modeling data set from each of the respective decision trees. In this approach, training and test data are randomly re-utilized in the construction of individual decision trees with an "out-of-bag" (oob) estimate of error rates equalling 20%. All factors in test populations were ranked by their relative importance (RI) in accurately classifying case and control study subjects.

### Pathways analysis

Data were analyzed using the Ingenuity Pathways Analysis (IPA) informatics platform (Ingenuity^® ^Systems, Redwood City, CA, USA). For univariate component analysis, the complete data set, including protein identifiers, corresponding quantitative expression and *P*-values was utilized. Each protein identifier was mapped to its corresponding gene object and overlaid onto a global molecular network developed from information contained in the IPA Knowledge Base. Networks of genes were then generated algorithmically based on their connectivity as established in the published literature. Fischer's exact test was used to calculate a *P*-value determining the probability that each biologic function and/or pathway assigned to the data set is due to chance alone.

In a separate analysis, plasma protein components identified as having high relative importance values in the RF multivariate analysis were used to explore putative biologic interactions using IPA Grow, Connect, and Path Explorer applications.

### Protein blot analysis

Plasma protein samples (30 μg each) from discordant twins and unrelated, matched controls were resolved by SDS-PAGE (10% precast Criterion gels, Bio-Rad, Hercules, CA, USA) and subsequently dry-blotted to PVDF membranes (iBlot system, Invitrogen, Carlsbad, CA, USA). Protein blots were blocked and incubated with rabbit polyclonal, primary antibodies recognizing human plasma PON1, RBP1, or LRG1 and transferrin (TF) as an internal control for 1 to 24 hours in TBS/0.05% Tween-20 (Abcam, Cambridge, MA, USA). Blots were washed and incubated for 30 minutes with a secondary antibody-HRP conjugate (goat anti-rabbit heavy and light chain IgG (Abcam)). Washed blots were incubated for one minute with chemiluminescent substrate and visualized using a GBOX HR50 molecular imaging system (Syngene, Frederick, MD, USA). Syngene GeneSnap imaging and analysis software was used to quantify and normalize replicate analyses of plasma protein levels.

## Results

### Plasma proteomic differential expression analysis

Many plasma proteins were differentially expressed similarly among multiple SAID as evidenced by comparisons of the discordant MZ twins and unrelated, matched controls (Table 1). Examinations of subjects stratified by diagnosis did not reveal any significant disease-specific alterations among these differentially expressed proteins (data not shown). Plasma proteomic profiles differentiating these three study groups comprised several functional categories including structural proteins, protease inhibitors, immune response-related (predominantly components of complement pathways), transporters, acute phase reactants, catalytic, coagulation and transcriptional factors (Table 1). As expected, the majority of plasma proteins identified were of extracellular origin (60 to 70%) while the remainder was derived from various subcellular compartments (for example, plasma membrane, cytoplasm and nucleus).

**Table 1:** Summary of significant differences detected (*P *< 0.05) among MZ twins discordant for SAID and unrelated, matched controls*

Affected vs. unaffected twins					
***Up-Regulated*:**	**Accession No**.	***P*-value**	**Exp. Ratio**	**Location**	**Function**
**α-1-antichymotrypsin precursor**	P01011	0.0008	1.37	Extracellular	Protease inhibitor
**Syntaxin 17**	P56962	0.0022	1.30	Plasma membrane	Vesicular transport
**Keratin type II**	P35908	0.0040	1.26	Cytoplasm	Structural
**Complement C8 precursor**	P07358	0.0070	1.14	Extracellular	Immune response
**α-1-acid glycoprotein**	P02763	0.0073	1.42	Extracellular	Acute phase reaction
**Retinoblastoma-binding protein 1**	P29374	0.0117	1.53	Nucleus	Transcriptional regulation
**Complement C4 precursor**	P01028	0.0122	1.36	Extracellular	Immune response
**Plasma protease C1 inhibitor precursor**	P05155	0.0144	1.19	Extracellular	Protease inhibitor
**Maltase-glucoamylase intestinal**	O43451	0.0167	1.51	Cytoplasm	Carbohydrate catabolism
**Hematopoietic-specific transmembrane-4 protein**	Q96HJ5	0.0197	1.21	Plasma membrane	Immune response
**Ubiquitin carboxyl-terminal hydrolase 29**	Q9HBJ7	0.0271	1.44	Unknown	Peptidase
**Complement C6 precursor**	P13671	0.0282	1.19	Extracellular	Immune response
**Complement C9 precursor**	P02748	0.0390	1.18	Extracellular	Immune response
**Kallikrein precursor**	P03952	0.0407	1.22	Extracellular	Peptidase
**Myomesin 1**	P52179	0.0418	1.27	Myofibers	Structural
**Leucine-rich α-2-glycoprotein precursor**	P02750	0.0473	1.26	Extracellular	pleiotropic**
***Down-Regulated:***					
**Serum paraoxonase/arylesterase 1**	P27169	0.0195	0.82	Extracellular	Detoxification/anti-oxidation
**Apolipoprotein A-II precursor**	P02652	0.0231	0.75	Extracellular	Transporter
**Fetuin-A**	P02765	0.0278	0.79	Extracellular	Protease inhibitor
**Minor histocompatibility antigen H13**	Q8TCT9	0.0356	0.78	Endoplasmic reticulum	Protease
Affected Twins vs. Unrelated, Matched Controls					
Up-Regulated:					
**Leucine-rich α-2-glycoprotein precursor**	P02750	0.0086	1.46	Extracellular	pleiotropic**
**Fibrinogen β-precursor**	P02675	0.0109	1.33	Extracellular	Coagulation
**Apolipoprotein C-IV precursor**	P55056	0.0155	1.69	Extracellular	Transporter
**Apolipoprotein E precursor**	P02649	0.0193	1.24	Extracellular	Transporter
**α-1-microglobulin/Inter-α-trypsin inhibitor**	P02760	0.0199	1.41	Extracellular	Protease inhibitor
**Nuclear receptor co-activator 6**	Q14686	0.0307	1.33	Nucleus	Transcriptional regulation
**Plasma retinol-binding protein precursor**	P02753	0.0356	1.30	Extracellular	Transporter
**Retinoblastoma-binding protein 1**	P29374	0.0361	1.43	Nucleus	Transcriptional regulation
**Apolipoprotein C-III precursor**	P02656	0.0395	1.29	Extracellular	Transporter
**Kallistatin precursor**	P29622	0.0461	1.19	Extracellular	Peptidase inhibitor
Down-Regulated:					
**Coagulation factor XII precursor**	P00748	0.0017	0.76	Extracellular	Coagulation
**Peroxisomal carnitine octanoyl transferase**	Q9UKG9	0.0196	0.67	Peroxisome	Metabolism fatty acid
**Ficolin 3 precursor**	NP_003656	0.0218	0.83	Extracellular	Immune response
**Fibrinogen C**	NP_116232	0.0248	0.83	Extracellular	Coagulation
**Probable ATP-dependent helicase DHX37**	Q8IY37	0.0253	0.89	Nucleus	RNA helicase
**Polycystin 1 precursor**	P98161	0.0341	0.78	Plasma membrane	Transporter/Signaling
**Complement C1q A chain precursor**	P02745	0.0350	0.69	Extracellular	Immune response
**NK cell receptor 3DL1 precursor**	P43629	0.0368	0.85	Plasma membrane	Immune response
**Serum paraoxonase/arylesterase 1**	P27169	0.0381	0.75	Extracellular	Detoxification/anti-oxidation
***Unaffected Twins vs. Unrelated, Matched Controls***					
***Up-Regulated:***					
**Apolipoprotein C-IV precursor**	P55056	0.0436	1.38	Extracellular	Transporter
**Apolipoprotein C-III precursor**	P02656	0.0493	1.24	Extracellular	Transporter
**Histone deacetylase 3**	O15379	0.0494	1.12	Nucleus	Chromatin modulation
Down-Regulated:					
**Alpha-1-antichymotrypsin precursor**	P01011	0.0036	0.84	Extracellular	Protease inhibitor
**Fibrinogen C**	NP_116232	0.0046	0.78	Extracellular	Coagulation
**Ficolin 3 precursor**	NP_003656	0.0049	0.80	Extracellular	Immune response
**Keratin type II**	P35908	0.0232	0.87	Cytoplasm	Structural
**Syntaxin 17**	P56962	0.0311	0.85	Plasma membrane	Vesicular transport
**Serum paraoxonase/arylesterase 1**	P27169	0.0466	0.69	Extracellular	Detoxification/anti-oxidation

*Abbreviations: No., number; Exp., expression

**protein-protein interaction; cell adhesion; signal transduction; granulocyte differentiation

**Figure 1:**
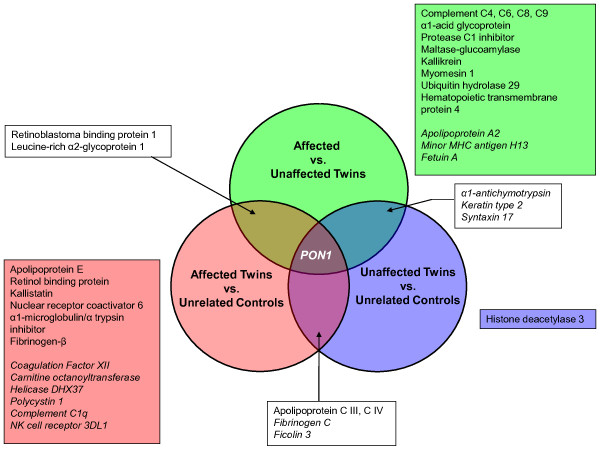
**Summary of protein inter-relationships**. Venn diagram depicting proteins detected at significantly different concentrations (*P *< 0.05) in plasma of monozygotic twins discordant for SAID and unrelated, matched controls. Pon1, paraoxonase 1

To illustrate these differential proteomic profiles, a Venn diagram depicting the inter-relationships of plasma protein profiles from each of the three two-group comparisons is shown in Figure 1. In this illustration, it is clear that comparisons of affected twins vs. either unaffected twins or unrelated, matched controls produced more complex profiles of differential protein expression relative to the comparison of unaffected twins vs. unrelated, matched controls. Relative to affected twins, it appears that the profile of unaffected twins more closely resembles that of unrelated, matched controls suggesting that disease status rather than genetic similarity between MZ twins might account for some differences in the number and magnitude of plasma protein levels detected differentially among the three study groups. A smaller number of proteins (α1 anti-chymotrypsin, type 2 keratin, and syntaxin 17) were the only protein markers shared uniquely among the discordant twin pairs.

In cases involving comparisons of affected twins to either unaffected twins or unrelated controls, multiple acute phase reactants and markers of immune activation are apparent. The PON1 gene product, paraoxonase 1, was the only marker exhibiting significant differences in expression levels in each of the three two-group comparisons (Figure 1). PON1 levels were reduced in the plasma of affected cases compared to either unaffected twins or unrelated, matched controls. Two additional markers, RBP1 and LRG1, were detected at modestly increased levels (approximately 1.2- to 1.5-fold) in affected twins compared to either unaffected twins or unrelated controls.

### Random Forest (RF) multivariate analyses

**Figure 2:**
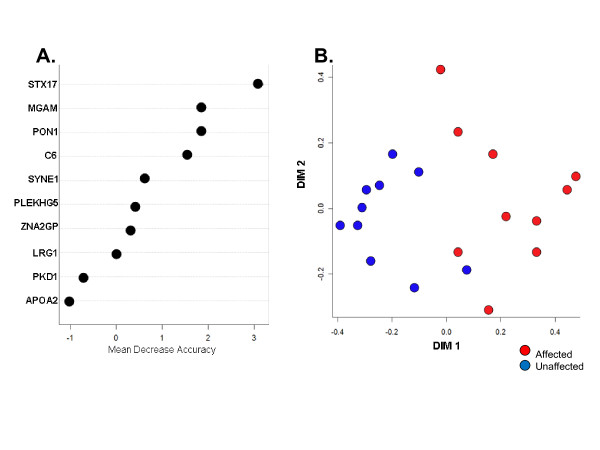
**Multivariate Random Forest analysis of protein components identified in plasma from MZ twins discordant for SAID**. **(A) **Relative importance values of individual protein components whose collective interactions in the RF model account for the effective stratification of affected vs. unaffected twins as described in Patients and Methods. **(B) **Cluster analysis of affected (red circles) and unaffected (blue circles) twins using the RF model described in (A). STX17, syntaxin; MGAM, α-glucosidase; PON1, paraoxonase 1; C6, complement component 6; SYNE1, spectrin repeat containing nuclear envelope 1; PLEKHG5, pleckstrin homology domain containing, family G, member 5; ZNA2GP, zinc-binding α-2-glycoprotein; LRG1, leucine-rich α-2-glycoprotein; PKD1, polycystic kidney disease-associated 1; APOA2, apolipoprotein A2

All identifiable protein markers for which differential quantitative data existed among subjects comprising the discordant twin study groups were analyzed by RF modeling to assess potential multivariate interactions. Among these, the top 50 protein markers exhibiting the strongest RI values for classifying accurately affected vs. unaffected twins were subsequently re-analyzed by RF using identical parameters. The resultant RF model classified correctly 90% of the 10 twin probands and 70% of the corresponding unaffected twins. The top 10 protein markers displaying the highest RI values for predictive classification are displayed in Figure 2A. Seven protein variables accounted for the majority of the predictive value of the model. Moreover, the four plasma protein markers with the highest RI scores (syntaxin 17 (STX17), maltase-glucoamylase (MGAM), paraoxonase 1 (PON1) and the sixth component of complement (C6)) were also significant in univariate analyses (see Table 1). An independent measure of the RF model examining the spatial proximity of test subjects produced a clear stratification of the affected and unaffected twin study groups (Figure 2B). These RF modeling data also suggest that assessing multiple, potentially interacting plasma protein factors might better define the proteomic profiles shared among multiple SAID.

### Pathway analysis

**Figure 3:**
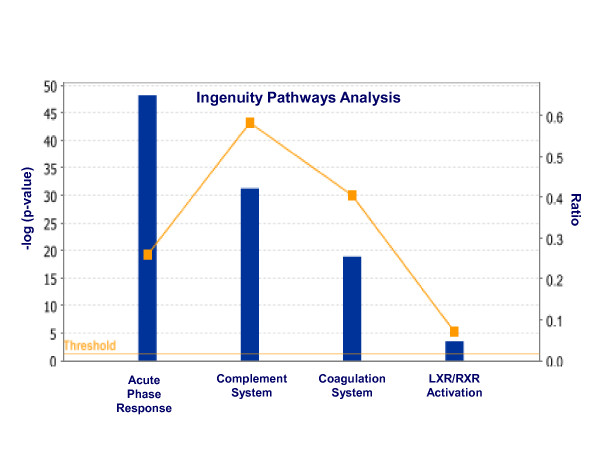
**Molecular pathway analysis**. Ingenuity Pathways Analysis was used to examine the differential expression values of the entire plasma protein datasets between SAID discordant MZ twins. Fischer's exact test was used to calculate a *P*-value determining the probability that the association between the markers in the dataset and the canonical pathway is attributable to chance alone (blue bars). The ratio of the number of genes from the dataset that map to a given pathway divided by the total number of markers that comprise the pathway is shown by the yellow line.

We performed molecular pathway analyses to assess if differential plasma protein levels detected in SAID compared to unaffected twins could be linked by common biologic pathways. Canonical pathways exhibiting the highest significance included mediators of the acute phase response to systemic inflammation (*P *= 6.7 × 10^-49^), complement fixation pathways (*P *= 5.2 × 10^-32^), coagulation system (*P *= 1.4 × 10^-19^) and retinoid receptor activation pathways (*P *= 3.2 × 10^-04^) (Figure 3). Similar differences were observed between comparisons of affected twins and unrelated, matched controls (data not shown).

**Figure 4:**
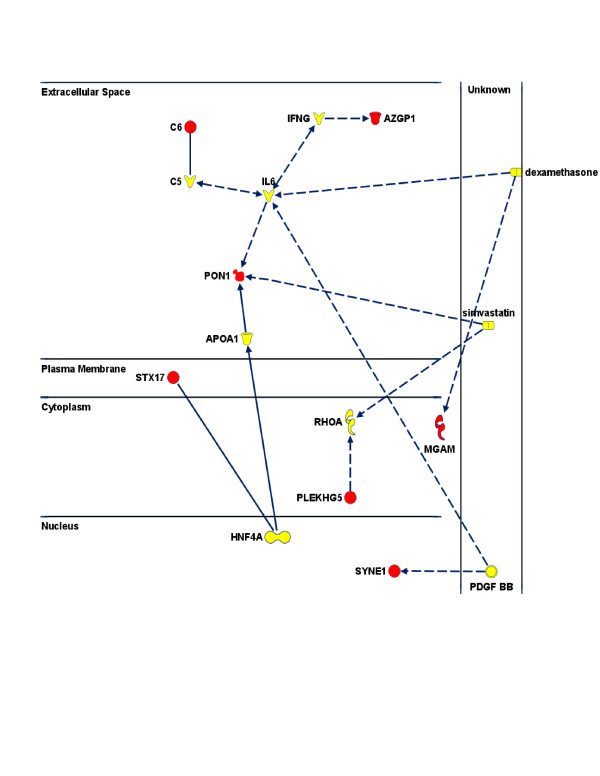
**Graphical representation of Ingenuity Pathways Analysis**. An IPA of research literature-based molecular relationships among protein components identified by multivariate Random Forest (RF) modeling (red) which describes possible interactions accounting for the accurate classification of affected vs. unaffected MZ twins discordant for SAID. The IPA Grow, Connect, and Path Explorer software functions were used to establish a spatial model utilizing the Shortest Pathway option. A minimum of two nodes (that is, interconnecting molecules), shown in yellow, were required to integrate the seven protein markers identified by RF analysis.

In a separate analysis, we examined those plasma proteins identified previously as having the highest RI scores for effectively classifying discordant twin pairs in a RF multivariate model. In this case, we utilized Ingenuity's Grow, Connect, and Path Explorer functions to examine putative molecular interactions and pathway integration among these candidate proteins (Figure 4). The shortest pathways by which the seven protein factors of interest (STX17, MGAM, PON1, C6, SYNE1, PLEKHG5 and AZGP1) were integrated required a minimum of two interconnecting nodes. For the majority of possible interactions, the PON1 gene product mapped as a central node connecting multiple protein factors identified by univariate and RF analyses. Many of the predicted PON1 interactions also involved the inclusion of the pro-inflammatory cytokine IL-6 as a secondary node integrating several other protein markers. The molecular pathways model illustrated in Figure 4 is representative of one of several possible means by which these candidate SAID markers might potentially interact.

### Protein blot analysis

**Figure 5:**
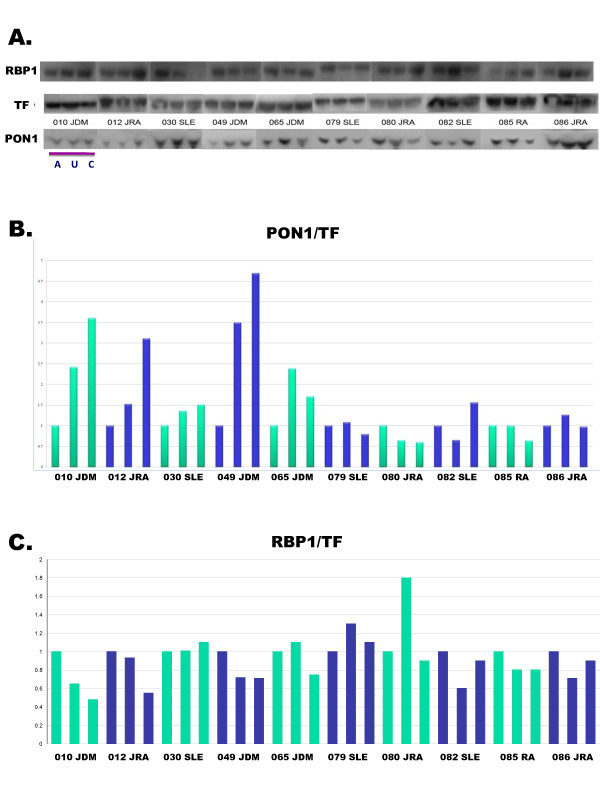
**Protein blot analysis of plasma PON1 and RBP1 levels from SAID discordant MZ twins and unrelated, matched controls**. **A**. Representative protein blot analyses of the RBP1 (140 kDa), TF (77 kDa) and PON1 (43 kDa) proteins for each of the 10 SAID-discordant twin pairs (A, affected twin; U, unaffected twin) and unrelated, matched controls (C). **B and C**. Summary of replicate blot assays illustrating plasma PON1 (B) and RBP1 (C) protein levels among SAID-discordant twins and unrelated, matched controls. Data were normalized to the constitutively expressed transferrin protein (PON1/TF and RBP1/TF, respectively) and plotted to compare relative differences in PON1 or RBP1 protein levels among the three study groups (affected twin, unaffected twin, and unrelated, matched control).

To assess further the potential significance of altered plasma PON1, RBP1, and LRG1 levels in SAID-affected twins, we evaluated each twin pair and corresponding unrelated, matched controls by protein blot analysis (Figure 5). As shown in Figure 5B, summary data for plasma protein levels of PON1 (approximately 43 kDa) and a transferrin (TF, approximately 77 kDa) normalization standard are illustrated for SAID-discordant twins and controls. A plot of PON1/TF values shows reduced plasma PON1 levels were observed for 5 out of the 10 independent twin pair/control sample sets irrespective of disease diagnosis (3 JDM, 1 JIA, and 1 SLE). A calculation of the mean reduction in PON1 levels among the 10 pairs of disease-discordant twins was similar in both protein blot and proteomics analyses (an approximate 1.2-fold reduction). A similar protein blot analysis of the RBP1 marker whose plasma levels were elevated in SAID-affected twins in comparisons with either unaffected twins or unrelated controls is shown in Figure 5C. Normalized plasma RBP1 levels (RBP1/TF) were increased approximately 1.2-fold in affected twins compared to unaffected twins or unrelated controls. A comparable increase of plasma RBP1 (approximately 1.45-fold) was detected in the proteomics analysis. We did not, however, detect elevated levels of LRG1 in SAID-affected twins by protein blot analysis in contrast to the approximately 1.4-fold increase observed by plasma proteomics (data not shown).

## Discussion

While certain autoimmune diseases share selected genetic, clinical and laboratory features, it is not clear if shared pathogenic mechanisms might link a number of SAID. One approach to the study of disease pathogenesis is the use of MZ twins as a means of controlling for the inherent genetic variability of study subjects in order to better assess the contribution of genetic, epigenetic and environmental factors [18]. MZ twins, however, are not genetically identical owing to various post-meiotic and age-related epigenetic modifications. Despite these differences, microarray analyses suggest that RNA expression levels of polymorphic genes are more tightly controlled in MZ twins than other first degree family members or unrelated controls [19,20].

In the present study, we have evaluated biologic pathways altered among multiple SAID by studying levels of plasma proteins using LC-ESI-MS from MZ twins discordant for SAID and unrelated, matched controls. Blood plasma is well-suited to the study of systemic or multi-organ diseases given its capacity to sample proteins from damaged tissues and detect changes in other physiologic pathways associated with complex host responses to disease processes and infectious and/or other environmental agents [11]. The human plasma proteome is one of the most complex and better characterized human bio-fluids wherein the identity and expression levels of its approximately 1,000 distinct protein constituents are currently cataloged [3].

Previous studies have examined human tissue and bio-fluid proteomes in autoimmune conditions with the goal of identifying disease-specific biomarkers to aid in improved disease diagnosis and understanding of underlying pathogeneses [4,21-29]. These findings point consistently to coordinated changes in the levels of multiple proteins involved in such canonical pathways as immune activation, signal transduction, cell adhesion, apoptosis, and acute phase responses, in addition to various transcription factors, structural and transport proteins. In fact, composite phenotypic profiles of coordinated changes in multiple protein factors and physiologic pathways rather than solitary biomarkers may prove more reliable in differentiating complex and sometimes overlapping autoimmune syndromes.

We examined multiple SAID in an attempt to uncover shared biomarkers or proteomic profiles, with the understanding that these otherwise heterogeneous disorders often share many clinical features, immunologic abnormalities, genetic risk factors and serum autoantibodies [30,31]. We hypothesized that certain proteomic profiles may be similar among patients with different SAID and that those profiles will differ from those of unaffected MZ twins. Moreover, we asked whether the proteomic profiles of unaffected twins more closely resembled that of unrelated, matched controls or possibly shared some features with their affected twins as a consequence of their genetic similarity and/or shared environmental exposures.

Collectively, our proteomics data from affected MZ twins was consisten with that from other published studies of human autoimmune diseases. Namely, the apparent coordinated regulation of multiple proteins from several canonical pathways (for example, immune regulation, acute phase response, protein and lipid homeostasis, apoptosis and signal transduction) appears to be associated with these chronic inflammatory conditions. In univariate analyses, we observed multiple proteins whose plasma levels were statistically different in affected twins compared to either unaffected twins or unrelated controls. Some of these proteins (for example, α1-microglobulin, fibrinogen, apolipoproteins A and E, complement C3 and C4B, and retinol binding protein) may exhibit altered plasma levels as a consequence of chronic inflammation as they were also reported as up-regulated in synovial fluid from osteoarthritis (OA) patients [23]. Increased levels of apolipoprotein A were also observed in isolated peripheral blood mononuclear cells from SLE patients and muscle biopsies of patients with inclusion body myositis [21,24,28]. Similarly, the leucine-rich α2 glycoprotein marker (LRG1) - a molecule involved in signal transduction, cell adhesion, and granulocyte differentiation - was elevated in plasma from our affected twins and was also found elevated in both the cerebrospinal fluid and serum proteomes from multiple sclerosis patients [26]. More recently, LRG1 was identified as a novel, serum pro-inflammatory biomarker for RA and Crohn's disease [32]. Molecular Pathways analysis of our total proteomics data set comparing SAID discordant MZ twins, helped us identify numerous acute phase reactants, immune complement components, coagulation factors, and retinol binding proteins as potentially important mediators of disease. Together, these data suggest that many of the physiological pathways altered in these patients are not necessarily disease-specific but rather may contribute to inflammatory processes shared by multiple SAID.

Proteomic data sets with large and complex arrays of candidate markers mapping across multiple biologic pathways present limits to the interpretation of univariate data by disregarding potential protein-protein interactions as a basis for accurate disease profiling. Investigators have employed machine learning algorithms for the multivariate analysis of large proteomic data sets derived from cancer prevention trials and human autoimmune disease studies [33,34]. Liu *et al*. described the use of a support vector machine algorithm to effectively classify RA patients and controls using serum proteomic component peaks [22]. Among the several decision tree ensemble methods available, we utilized the Random Forests algorithm to create a model which accurately classified affected vs. unaffected twin pairs. Putative interactions among seven proteins (STX17, MGAM, PON1, C6, SYNE1, PLEKHG5 and AZGP1) accounted for the majority of this effect. Several of these proteins were likewise identified in our univariate analyses (STX17, MGAM, PON1 and C6). The STX17 marker was one of three proteins whose altered plasma levels was unique to the comparison of discordant MZ twins, while PON1 was the only marker identified with statistically different levels in each of the three two-group comparisons.

The PON1 gene product, paraoxonase 1, is an arylesterase that serves an important role in several physiological pathways including the detoxification of xenobiotics - most notably organophosphorus metabolites associated with pesticide exposures - as well as reducing oxidative damage when associated with circulating high and low density lipoproteins [35-37]. Interestingly, functional polymorphisms in the PON1 gene influence expression levels and activity of the enzyme and have been associated with several immune-mediated conditions, atherosclerotic risk, and possibly influence responses to anti-TNF-α therapy in RA [38-41].

Several independent lines of evidence implicate reduced plasma PON1 levels as a potential biomarker for a subset of SAID [39,42,43]. In our present study, we observed an apparent gradient of decreasing PON1 levels among our three study groups in univariate analyses whereby PON1 levels were lowest in SAID-affected twins and highest in unrelated controls. Also, PON1 was identified as an informative marker in a multivariate RF model, which effectively segregated SAID affected vs. unaffected twins. In molecular pathway modeling, PON1 mapped as a central node in interactions predicted among all the relevant factors in the RF analysis. More recently, certain PON1 polymorphic variants were implicated as risk factors for other chronic inflammatory diseases, including RA and types 1 and 2 diabetes [44,45]. Plasma protein blot analysis of our twin pairs and matched, unrelated controls demonstrated reduced plasma PON1 levels in 50% of the twin cases independent of disease phenotype. We speculate that shared or similar environmental factors, such as pesticide exposures, might influence the development of different SAID by a common mechanism [46].

There are several limitations to our plasma proteomics study design. Most importantly, small sample sizes and the resulting decrease in statistical power owing to the difficulties associated with the identification and recruitment of SAID-discordant MZ twins with recent disease onset. Also, the heterogeneity of human study subjects, including variations in environmental exposures, clinical phenotypes, disease activity and duration and immunosuppressive therapies may influence plasma protein composition and present potential confounders. Additionally, given the capacity of mass spectrometric techniques to detect several thousand component peaks from individual plasma samples, higher false discovery rates (FDR) are anticipated in the absence of corrections for multiple statistical comparisons. Despite these limitations, most of the candidate markers and molecular pathways identified in our study are consistent with those identified in other studies of individual human autoimmune disease [21-28,44,47].

## Conclusions

We have described proteomic profiles common to multiple, different SAID. We analyzed SAID-discordant MZ twins to minimize polymorphic gene effects and found that, in comparison to affected twins, plasma proteomes of unaffected twins more closely resemble those of unrelated, matched controls. These data suggest that in addition to genetic predispositions, disease pathogenesis in MZ twins who develop SAID are likely influenced by post-meiotic genetic events (for example, copy number variations between MZ twins), different epigenetic modifications, epistatic protein interactions, and/or environmental exposures that promote pro-inflammatory biologic pathways. Moreover, the use of complex proteomic profiles - rather than individual biomarkers - may provide a more highly integrated description of immune dysfunction and disease pathogeneses. Our hope is that such studies might lead to earlier and more accurate diagnostics, and more effective, targeted therapeutics.

## Abbreviations

C6: sixth component of complement; CV: coefficients of variation; EM: expectation-maximization; FDR: false discovery rates; IPA: Ingenuity Pathways Analysis; LC-ESI-MS: liquid chromatography electrospray ionization mass spectrometry; MGAM: maltase-glucoamylase; MS/MS: tandem mass spectrometry; MZ: monozygotic; OA: osteoarthritis; oob: "out-of-bag"; Pon1: paraoxonase 1; RF: random forest; RI: relative importance; SAID: systemic autoimmune disease; SLE: systemic lupus erythematosus; TF: transferrin; TOF: time-of-flight

## Competing interests

The authors declare that they have no competing interests.

## Authors' contributions

TO'H prepared the samples, performed the RQ-PCR studies, data and bioinformatic analyses and wrote the manuscript. ZL carried out the protein blot analyses and data analysis, and prepared and edited the manuscript. LR took part in patient recruitment, clinical assessments and data analyses, and manuscript editing. LG performed protein blot analyses, data analysis, and edited the manuscript. MG took part in patient recruitment, clinical assessments and manuscript editing. FM participated in study design, patient recruitment, clinical assessments, data analyses, and in manuscript preparation and editing.

## Supplementary Material

Additional file 1 **Summary of differential plasma protein levels in comparisons of SAID-affected twins vs. unrelated, matched controls**. Excel file documenting all processed, primary data for individual plasma proteins levels in the respective comparison groups as determined by LC-ESI-MS with corresponding statistical analyses (see Materials and methods). Click here for file

Additional file 2 **Summary of differential plasma protein levels in comparisons of SAID-affected twins vs. said-unaffected twins**. Excel file documenting all processed, primary data for individual plasma proteins levels in the respective comparison groups as determined by LC-ESI-MS with corresponding statistical analyses (see Materials and methods). Click here for file

Additional file 3 **Summary of differential plasma protein levels in comparisons of SAID-Unaffected twins vs. unrelated, matched controls**. Excel file documenting all processed, primary data for individual plasma proteins levels in the respective comparison groups as determined by LC-ESI-MS with corresponding statistical analyses (see Materials and methods). Click here for file
